# Pain severity and quality of life among patients undergoing hemodialysis

**DOI:** 10.7717/peerj.21892

**Published:** 2026-11-12

**Authors:** Khulud Adel Abudawood, Ruba Albeladi, Ruqayah Alhindi, Ethar Alqarni, Nouf Aljelaidy, Reem Alghamdi, Majd Alfahmi, Raghad Alkathiri, Nada Almasri, Reham Alotaibi, Fatmah Ahmed Alamoudi

**Affiliations:** 1 Ministry of National Guard Health Affairs, Jeddah, Saudi Arabia; 2 College of Nursing, King Saud bin Abdulaziz University for Health Sciences, Jeddah, Saudi Arabia; 3 King Abdullah International Medical Research Center, Jeddah, Saudi Arabia; 4 Medical Surgical Nursing Department, Prince Sultan Military College of Health Sciences, Dhahran, Saudi Arabia

**Keywords:** Hemodialysis, Chronic kidney disease, Pain, Quality of life

## Abstract

**Objectives:**

Chronic kidney disease (CKD) is a progressive condition that imposes physical and psychological burdens, negatively affecting the quality of life (QOL) of patients undergoing hemodialysis (HD). Pain is a prevalent symptom linked to HD treatment. Multiple sociodemographic and clinical factors affect the QOL of people with CKD; therefore, healthcare providers must understand the relationship between these variables. This study aimed to examine the relationship between pain severity and QOL among adult patients undergoing hemodialysis.

**Methods:**

An observational correlational cross-sectional study was conducted at inpatient and outpatient HD units. A convenience sample of 157 adult patients participated. They completed a sociodemographic questionnaire, the Arabic Short-Form McGill Pain Questionnaire (SF-MPQ), and the World Health Organization Quality of Life–BREF (WHOQOL-BREF).

**Results:**

Participants reported a low-to-moderate QOL across domains, with the psychological domain having the lowest mean (55.04 ± 17.84). The mean pain severity was 3.94 ± 3.34, with 15% of the sample experiencing severe pain. Significant negative correlations were found between pain severity and physical (r = −0.444, *p* < 0.001), psychological (r = −0.263, *p* < 0.001), and environmental (r = −0.218, *p* = 0.006) QOL domains. Regression analysis identified monthly income and age as significant predictors of psychological, social, and environmental QOL. Additionally, hemodialysis-specific factors showed limited predictive power, though longer dialysis sessions predicted better psychological QOL (*p* = 0.005) and higher weekly dialysis frequency predicted better overall QOL (*p* = 0.007).

**Conclusions:**

Pain adversely affects multiple aspects of QOL in HD patients. Comprehensive interventions focusing on pain management and socioeconomic support are essential to improve QOL outcomes in this adult population.

## Introduction

Chronic kidney disease (CKD) is a global health problem affecting more than 800 million people, or 10% of the total population (Kovesdy, 2022). This has led to an increase in the mortality rate and financial burden on healthcare systems (Higuita-Gutiérrez, Velasco-Castaño & Jiménez Quiceno, 2019). According to the World Health Organization, the CKD mortality rate has reached 1.3 million, ranking as the tenth leading cause of death worldwide (World Health Organization, 2020). There is a significant financial burden on the healthcare system due to the high cost of CKD treatment, estimated at an annual average of $57,334 (Jha et al., 2023). Thus, individuals with low socioeconomic status often cannot afford high-quality or comprehensive healthcare for CKD management (Kefale et al., 2019).

According to the Saudi Arabia Ministry of Health (2021), the total number of patients undergoing hemodialysis (HD) has reached 20,000, with an estimated CKD incidence of 9,892 per 100,000 people. While HD remains the most common and effective treatment for individuals with CKD to manage physical disease complications, it introduces complex complications that negatively affect patients’ QOL through thrice-weekly treatment, dietary restrictions, and medication regimens (Yonata et al., 2022). Because CKD imposes a heavy symptom burden, adult patients undergoing HD represent a vulnerable group affected by treatment-related complications and reduced quality of life (QOL).

QOL, as defined by the WHO, is “an individual’s perception of their position in life in the context of the culture and value systems in which they live and concerning their goals, expectations, standards and concerns.” QOL is compromised in CKD patients across physical, psychological, social, and environmental aspects. Several studies have shown that people on HD report significantly lower QOL than the general population (Al Salmi et al., 2021; Kefale et al., 2019).

Pain is highly prevalent, affecting 33% to 82% of HD patients across various studies (Brkovic, Burilovic & Puljak, 2016). Common sources of pain in this population include procedure-related pain from arteriovenous fistula cannulation, intradialytic muscle cramps, headaches, musculoskeletal discomfort, and pain associated with prolonged immobility during sessions (Ibrahim, Badawi & Abdelaal, 2022; Rodrigues & Costeira, 2024). Older patients on HD may experience more severe or persistent pain due to age-related physiological changes and comorbidities. Higher pain severity is consistently correlated with lower QOL across all domains (Samoudi et al., 2021; Ishtawi et al., 2023). Physically, anemia, edema, muscle cramps, and pain limit mobility and impair QOL (Pretto et al., 2020). Psychologically, higher pain levels contribute to stress, depression, and anxiety (Masià-Plana et al., 2022).

Socially and spiritually, strong support networks and religiosity significantly enhance well-being (Asiri et al., 2023). The demanding treatment schedule limits time available for social activities and may force patients to retire early or lose their jobs. Financial and transportation barriers further create substantial economic challenges that affect patients’ and their families’ well-being (Bayoumi et al., 2013). Multiple demographic and clinical variables, such as age, gender, income, education, and employment status, influence QOL outcomes among HD patients. Social support and religiosity have emerged as protective factors that improve coping and enhance psychological well-being, suggesting that patients with strong familial and spiritual connections demonstrate greater adaptation to treatment plans (Cruz et al., 2017; Abbas & Ali, 2023).

Despite the growing interest in studying QOL among HD patients, it is essential to explore the specific factors influencing their pain and QOL outcomes comprehensively. Region-specific studies remain scarce in the Middle East, where cultural and religious contexts may strongly influence both pain perception and QOL (Al Salmi et al., 2021). Therefore, this study aimed to examine the relationship between pain severity and QOL among adult patients with CKD undergoing hemodialysis in Saudi Arabia. The results will guide evidence-based nursing interventions designed to improve pain outcomes and holistic QOL.

## Materials and Methods

### Design

This study utilized an observational correlational cross-sectional design.

### Setting and sample

The study was conducted in the inpatient and outpatient HD units at King Abdulaziz Medical City (KAMC) in Jeddah, Saudi Arabia. A nonprobability convenience sampling technique was used. The inclusion criteria were as follows: (1) adult patients (aged 18 years or older) with a confirmed CKD diagnosis; (2) currently receiving HD treatment; (3) current experience of pain; (4) ability to communicate in Arabic or English; and (5) provision of written informed consent. Patients with cognitive, mental, or neurological conditions affecting their ability to understand or complete the questionnaires were excluded. A total of 157 adult patients participated in the study.

### Ethical considerations

Ethical approval was obtained from the Institutional Review Board (IRB) at King Abdullah International Medical Research Center (KAIMRC) (IRB# SP23J/165/11), as well as from the participating HD units. Participation was strictly voluntary, and written informed consent was secured from all participants. No identifiable personal information was collected, ensuring anonymity and confidentiality.

### Data collection procedures

Data were collected from December 2023 to February 2024 *via* structured questionnaires. Surveys were distributed electronically *via* Google Forms during HD sessions. Trained research assistants were available to assist participants with reading or clarifying items if requested, taking care not to direct or influence their responses. This assistance ensured the inclusion of patients with varying literacy levels or limited physical dexterity due to vascular access.

## Materials

The data were collected *via* a structured questionnaire composed of three main sections:

- **Sociodemographic and Clinical Data:** This section assessed age, gender, nationality, marital status, education level, monthly income, employment status, smoking status, and comorbidities. Additionally, hemodialysis-related characteristics were collected, including years since HD diagnosis, chronic kidney disease (CKD) stage, frequency of HD sessions per week, and dialysis session duration.
- **Pain Assessment:** Pain was assessed using the Arabic version of the Short-Form McGill Pain Questionnaire (SF-MPQ) (Melzack, 1987; Terkawi et al., 2017). For the purpose of this analysis, pain severity was measured using the self-reported 0–10 intensity rating obtained during the hemodialysis session when the questionnaire was completed. The Arabic SF-MPQ has demonstrated good reliability in previous studies, with a Cronbach’s alpha of 0.70–0.89 and an intraclass correlation coefficient (ICC) of 0.71.
- **Quality of Life:** QOL was measured using the Arabic version of the World Health Organization Quality of Life–BREF (WHOQOL-BREF). This 26-item instrument covers four domains: physical health, psychological well-being, social relationships, and environment, along with two global items assessing overall QOL and satisfaction with health status. The Arabic version has shown satisfactory internal consistency, with Cronbach’s alpha values ranging from 0.70 to 0.85 (Ohaeri & Awadalla, 2009).

### Data analysis

Data were analyzed using SPSS version 25. Descriptive statistics were used to summarize sample characteristics and variable distributions. Pearson correlations were applied to examine relationships between pain severity, age, and QOL domains. Multiple linear regression models identified sociodemographic predictors of QOL domains. Separate regression models were run to examine the predictive value of hemodialysis-specific characteristics on QOL outcomes. Statistical significance was set at *p* < 0.05.

## Results

A total of 157 adult patients undergoing HD were included. Patient ages ranged from 20 to 80 years, with a mean age of 50.04 ± 13.75 years. The sample was divided into the following age groups: ≤24 years (*n* = 5, 3.0%), 25–44 years (*n* = 49, 31.2%), 45–64 years (*n* = 78, 49.7%), and ≥65 years (*n* = 25, 15.9%). Approximately half of the participants were male (*n* = 79, 50.3%) and female (*n* = 78, 49.7%). The majority were Saudi nationals (*n* = 140, 89.2%) and married (*n* = 103, 65.6%). Educational attainment included elementary school (*n* = 49, 31.2%), secondary school (*n* = 20, 12.7%), high school (*n* = 48, 30.6%), and diploma or higher (*n* = 40, 25.5%).

Employment rates were low, with 80.3% (*n* = 126) of participants unemployed. Regarding monthly income, the majority (*n* = 100, 63.7%) earned <5,000 SR, 21.0% (*n* = 33) earned 5,000–10,000 SR, 7.6% (*n* = 12) earned 10,000–15,000 SR, and 7.6% (*n* = 12) earned >15,000 SR. The majority were nonsmokers (*n* = 135, 86.0%). A significant proportion (*n* = 130, 82.8%) had existing comorbidities, primarily hypertension (*n* = 110, 70.1%), diabetes mellitus (*n* = 57, 36.3%), heart disease (*n* = 30, 19.1%), and others (*n* = 31, 19.7%) (Table 1).

**Table 1:** Sociodemographic and clinical characteristics of the participants.

Variable	Frequency (*n*)	Percentage (%)	(Mean ± SD)
Age			50.04 (13.75)
Age groups			
≤24	5	3%	
25–44	49	31.2%	
45–64	78	49.7%	
≥65	25	15.9%	
Gender			
Female	78	49.7	
Male	79	50.3	
Nationality			
Saudi	140	89.2	
Non-Saudi	17	10.8	
Education levels			
Elementary school	49	31.2	
Secondary school	20	12.7	
High school	48	30.6	
Diploma or higher	40	25.5	
Marital status			
Single	54	34.4	
Married	103	65.6	
Employment			
Employed	31	19.7	
Unemployed	126	80.3	
Monthly income			
<5,000 SR	100	63.7	
5,000–10,000 SR	33	21.0	
10,000–15,000 SR	12	7.6	
>15,000 SR	12	7.6	
Smoking			
No	135	86.0	
Yes	22	14.0	
Comorbidity			
No	27	17.2	
Yes	130	82.8	
Hypertension	110	70.1	
Diabetes mellitus	57	36.3	
Heart disease	30	19.1	
Others	31	19.7	

**Note:**

SR, Saudi Riyal; SD, standard deviation.

The QOL of participants was assessed across four domains. Overall, QOL was low to moderate, with the psychological domain yielding the lowest mean score (55.04 ± 17.84). The physical health domain had a mean of 62.83 ± 20.39. Higher scores were observed in the environment (70.54 ± 18.53) and social relationships (70.44 ± 22.52) domains. The overall QOL mean score was 71.66 ± 24.85, and satisfaction with health status was 74.84 ± 27.08 (Table 2).

**Table 2:** Descriptive statistics for QOL domains.

QOL domains	Minimum	Maximum	Mean	SD
Physical health	10.71	100.00	62.83	20.39
Psychological	8.33	79.17	55.04	17.84
Social relationships	0	100.00	70.44	22.52
Environment	18.75	100.00	70.54	18.53
Overall quality of life	0	100.00	71.66	24.85
Satisfaction with health status	0	100.00	74.84	27.08

**Note:**

QOL, Quality of life; SD, standard deviation.

The mean pain severity was 3.94 ± 3.34 on the 0–10 scale. The pain severity distribution was as follows: 25% minimal/no pain, 35% mild, 25% moderate, and 15% severe. Regarding physical health items, 45.2% (*n* = 71) reported that pain prevented them from doing daily activities. Additionally, 47.8% (*n* = 75) reported a moderate to extreme need for medical treatment to function daily (M = 2.41, SD = 1.33). The lowest score in the physical domain was having enough energy for everyday life (Q10: M = 3.00, SD = 1.25). Satisfaction with sleep and rest (Q16) had a mean of 3.61 (SD = 1.26).

In the psychological domain, satisfaction with oneself (Q19) scored high (M = 4.20, SD = 1.04), with 82.8% (*n* = 130) satisfied or very satisfied. The frequency of negative feelings (Q26) was relatively low (M = 2.16, SD = 1.13). Within social relationships, satisfaction with personal relationships (Q20) and support from friends (Q22) had means of 4.10 (SD = 1.09) and 4.03 (SD = 1.19), respectively. However, satisfaction with sexual activity (Q21) was notably low at 22.3% (*n* = 35). For the environment domain, access to health services (Q24) had the highest mean score (M = 4.34, SD = 0.94), with 54.1% (*n* = 85) very satisfied. Satisfaction was also high for transportation (Q25: M = 4.06, SD = 1.02) and living conditions (Q23: M = 3.96, SD = 1.06). In contrast, having enough money to meet needs (Q12) had a lower mean (M = 3.32, SD = 1.28), with 28.0% (*n* = 44) reporting having little or not at all enough money. Opportunities for leisure activities (Q14) were similarly limited (M = 3.23, SD = 1.26), with 28.7% (*n* = 45) reporting limited or no chance.

Correlation analysis indicated that greater pain severity was significantly and negatively associated with the physical (r = −0.444, *p* < 0.001), psychological (r = −0.263, *p* < 0.001), and environmental (r = −0.218, *p* = 0.006) QOL domains, as well as satisfaction with health status (r = −0.182, *p* = 0.022). Age was significantly positively associated with the psychological (r = 0.284, *p* < 0.001) and environmental (r = 0.234, *p* = 0.003) domains (Table 3).

**Table 3:** Correlations between pain severity, age, and QOL domains.

QOL domians	Age		Pain severity	
	r	*p* value	r	*p* value
Physical health	−0.129	0.109	−0.444	<0.001*
Psychological	0.284	<0.001*	−0.263	<0.001*
Social relationships	0.132	0.099	−0.130	0.103
Environment	0.234	0.003*	−0.218	0.006*
Overall quality of life	−0.129	0.107	−0.022	0.788
Satisfaction with health status	−0.137	0.087	−0.182	0.022*

**Notes:**

QOL, quality of life.

* *p* < 0.05.

Sociodemographic regression analysis identified that monthly income was a significant positive predictor for psychological health (B = 6.449, *p* < 0.001), social relationships (B = 6.101, *p* = 0.014), environment (B = 9.227, *p* < 0.001), and satisfaction with health (B = 6.946, *p* = 0.020) (Fig. 1). Age positively predicted psychological health (B = 0.293, *p* = 0.016) and environment (B = 0.242, *p* = 0.044), but negatively predicted satisfaction with health (B = −0.592, *p* = 0.002) (Fig. 2). Nationality (non-Saudi) negatively predicted social relationships (B = −11.972, *p* = 0.042) (Fig. 3), and being married positively predicted satisfaction with health (B = 11.392, *p* = 0.021) (Table 4) (Fig. 4).

**Figure 1:**
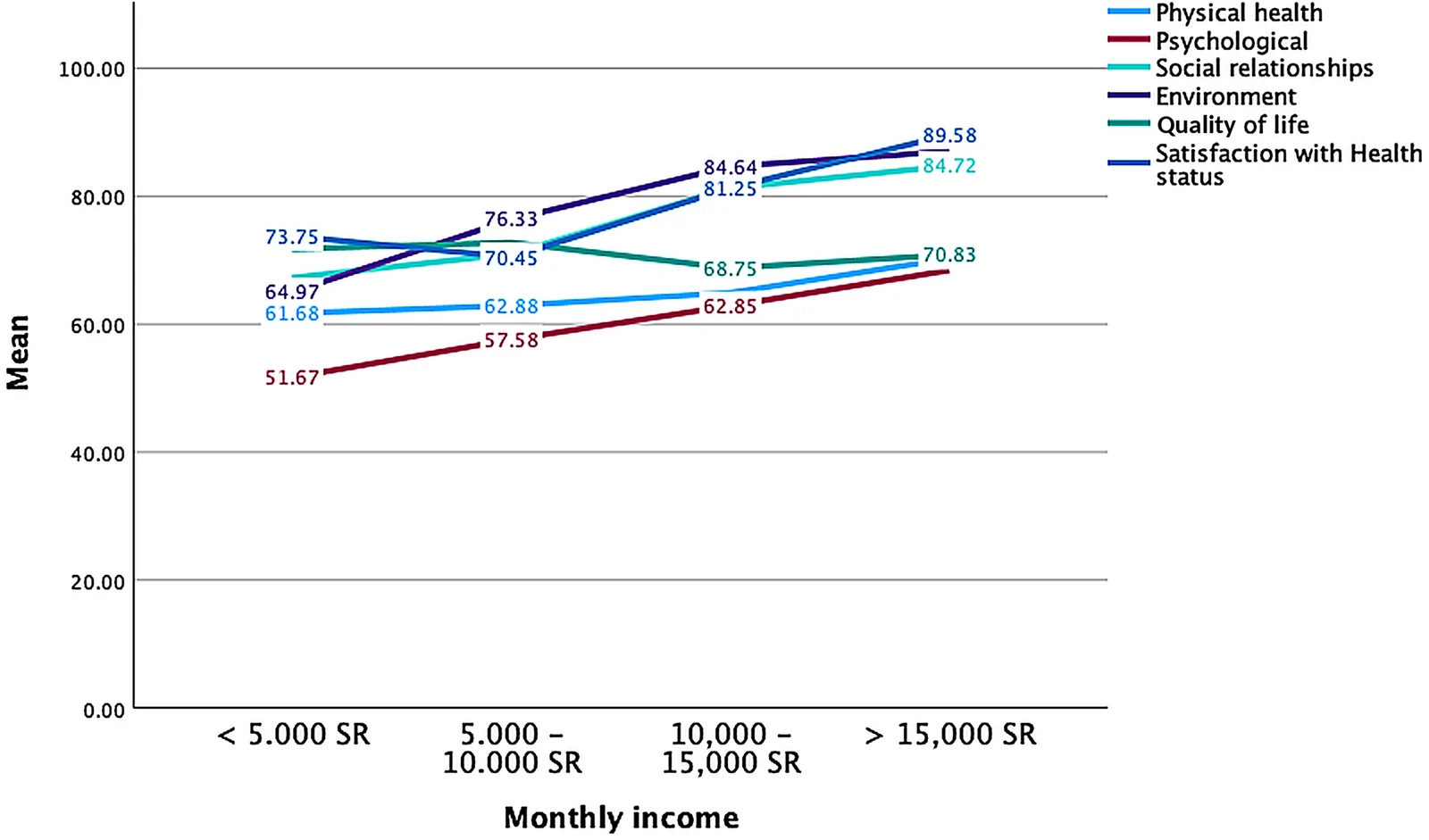
Mean differences between age groups and quality-of-life domains.

**Figure 2:**
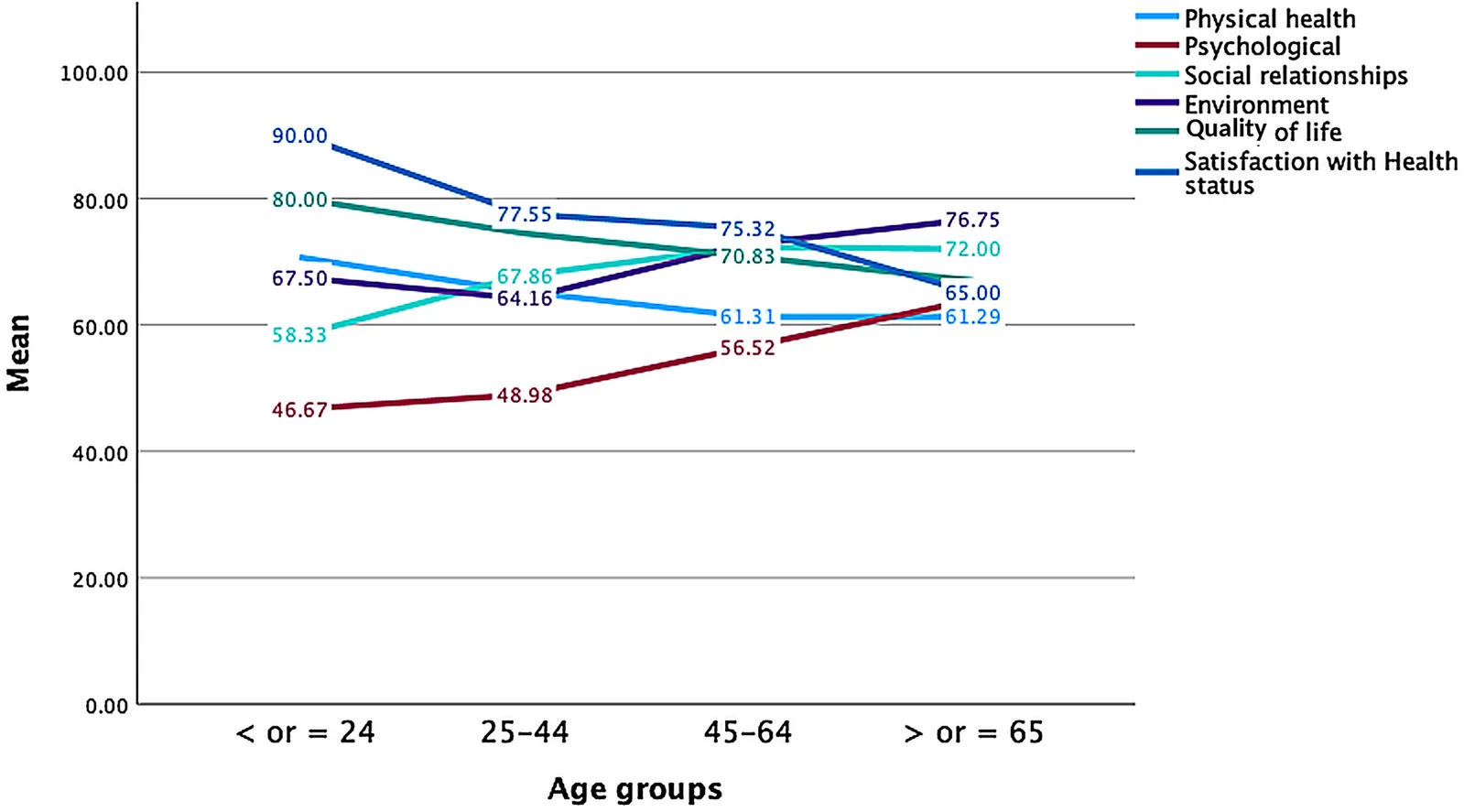
Mean difference between monthly income and quality of life domains.

**Figure 3:**
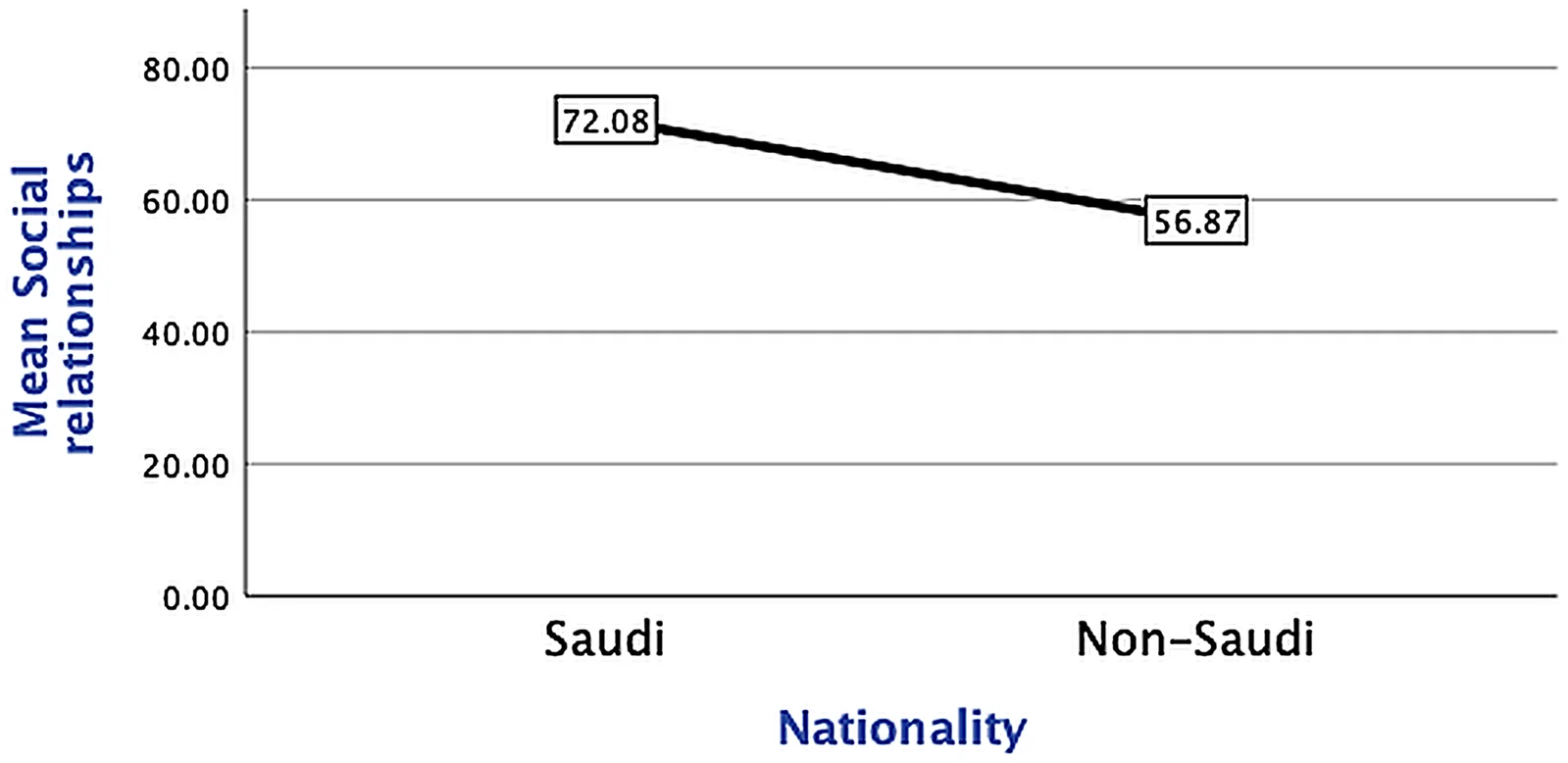
Mean differences between the nationality and social relationships quality of life domain.

**Table 4:** Sociodemographic variables and significant predictors for QOL domains.

QOL domains	R^2^	F (df1, df2)	*p* (model)	Significant predictors	B (unstandardized coefficient)	*p* (predictor)	Direction of association
Physical health	0.041	0.625 (10, 146)	0.791	None	—	—	—
Psychological health	0.169	2.979 (10, 146)	0.002	Monthly income	6.449	<0.001	Positive
				Age	0.293	0.016	Positive
Social relationships	0.141	2.403 (10, 146)	0.011	Monthly income	6.101	0.014	Positive
				Nationality	−11.972	0.042	Negative
Environment	0.242	4.672 (10, 146)	<0.001	Monthly income	9.227	<0.001	Positive
				Age	0.242	0.044	Positive
Overall quality of life	0.066	1.037 (10, 146)	0.416	None	—	—	—
Satisfaction with health	0.136	2.295 (10, 146)	0.016	Monthly income	6.946	0.02	Positive
				Marital status	11.392	0.021	Positive
				Age	−0.592	0.002	Negative

**Note:**

QOL, quality of life.

**Figure 4:**
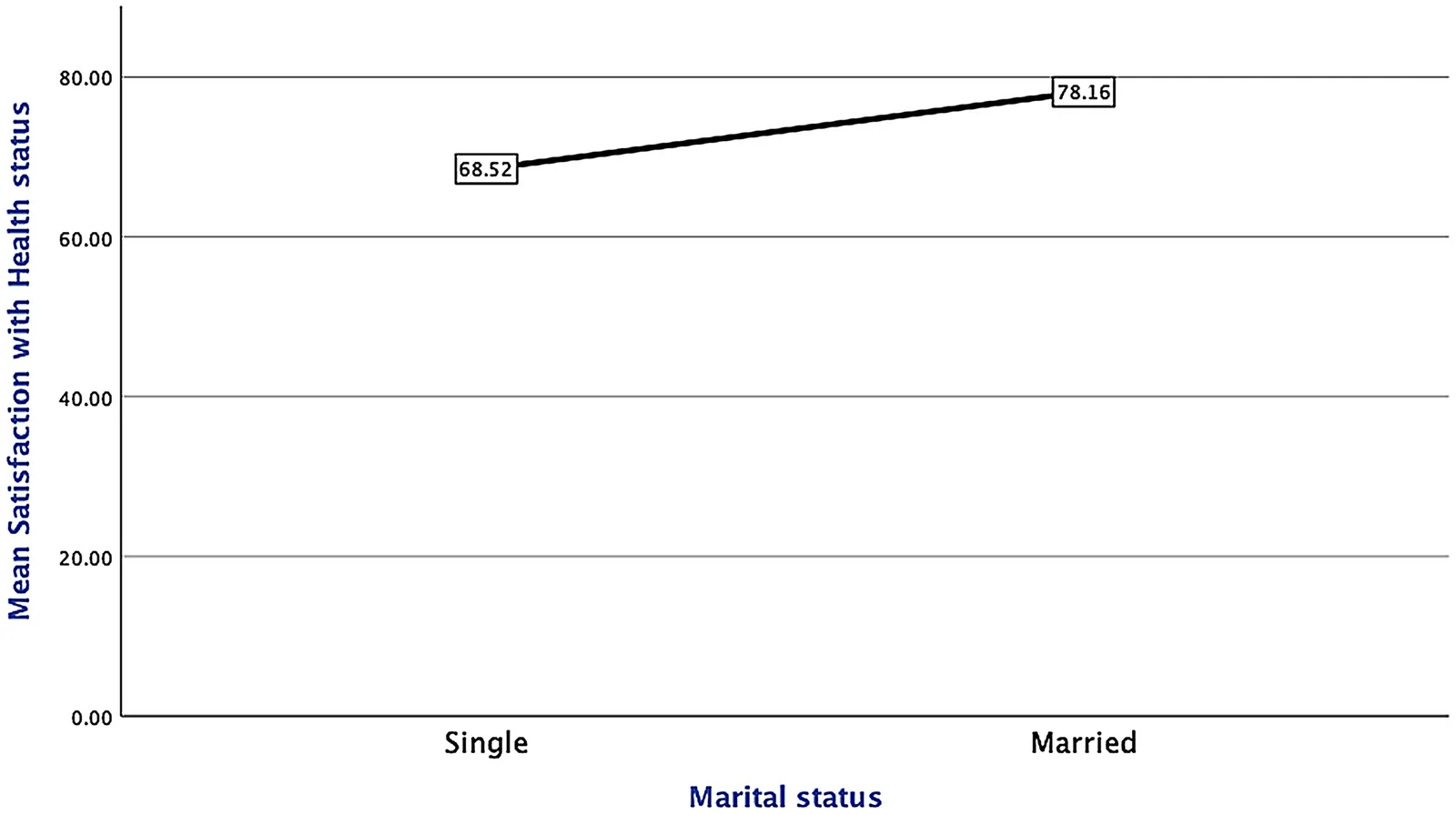
Mean differences in marital status and satisfaction with the health status quality of life domain.

In addition to sociodemographic variables, we analyzed hemodialysis-specific clinical characteristics among the cohort (Table 5). More than half of the patients (57.3%) had been diagnosed with end-stage renal disease for 1–6 years. Notably, 86.0% of the participants were unaware of their specific CKD stage, and 98.7% received hemodialysis three times per week.

**Table 5:** Hemodialysis-specific clinical characteristics.

Characteristic	Category	*n*	%
Years since HD diagnosis	1–6 years	90	57.3
Years since HD diagnosis	>6 years	67	42.7
CKD stage	Stage 2	1	0.6
CKD stage	Stage 4	3	1.9
CKD stage	Stage 5	18	11.5
CKD stage	Unknown/I do not know	135	86.0
Hemodialysis sessions frequency per week	3 times/week	155	98.7
Hemodialysis frequency per week	<3 times/week	2	1.3

Regression analysis evaluating these hemodialysis-specific factors (Table 6) demonstrated that longer dialysis session durations positively predicted psychological QOL scores, while higher weekly dialysis frequencies were associated with higher overall QOL scores. However, the overall variance explained by these clinical models remained low.

**Table 6:** Hemodialysis-related predictors of quality of life.

QOL outcome	Predictor	B	SE	t	*p* value
Psychological domain	Dialysis session duration (hours)	1.29	0.45	2.84	0.005
Overall quality of life	Hemodialysis frequency per week	47.82	17.52	2.73	0.007

## Discussion

The findings of this study emphasize the multifaceted factors influencing the QOL of HD patients in Saudi Arabia. Pain severity emerged as a critical determinant, demonstrating significant negative impacts on physical health, psychological well-being, and environmental QOL. This finding reinforces the need for effective pain management as a cornerstone of QOL interventions for HD patients (Pretto et al., 2020; Rodrigues & Costeira, 2024). Nearly half of the participants reported that pain interfered with their daily functioning, which aligns with previous research identifying pain as a significant barrier to physical well-being (Brkovic, Burilovic & Puljak, 2016; Samoudi et al., 2021). Similarly, the high proportion of individuals requiring medical treatment to maintain daily functioning and reporting low energy levels align with literature emphasizing fatigue and treatment dependency as core challenges in CKD (Fletcher et al., 2022).

In Saudi Arabia, cultural attitudes toward pain and coping mechanisms differ from those in Western contexts. Many patients rely on religious faith and spiritual practices, which may mitigate the psychological burden of chronic disease (Cruz et al., 2017; Asiri et al., 2023). However, studies highlight potential gaps in the availability of specialized pain management programs for HD patients in the region (Al Wakeel et al., 2012). This indicates a critical need for comprehensive programs combining pharmacological treatments with culturally tailored non-pharmacological interventions.

The study also revealed a positive association between age and both the psychological and environmental domains, suggesting that older patients in this adult cohort may possess greater psychological resilience or adaptability to their environment, possibly due to accumulated life experience and established family support structures (Abbas & Ali, 2023). In the local context, strong family involvement heavily influences psychological well-being, and patients with robust community support often report significantly higher psychosocial QOL compared to those with limited networks (Asiri et al., 2023).

Monthly income was a consistent predictor across multiple domains, positively influencing psychological health, social relationships, environmental QOL, and health satisfaction. Patients with higher incomes likely experience less financial stress and greater access to health resources, enhancing their ability to cope with HD demands (Al Wakeel et al., 2012; Bayoumi et al., 2013). Socioeconomic disparities, particularly among low-income populations facing logistical barriers to accessing services, emphasize the importance of financial support programs to improve psychological and physical well-being.

Regarding hemodialysis-specific characteristics, our analysis yielded mixed results. We found that longer dialysis session duration was associated with better psychological QOL scores. Additionally, higher weekly dialysis frequency was associated with better overall QOL. However, these findings must be interpreted with caution. As noted, the explanatory power of these models was low (R^2^ ≤ 0.05), and there was very little variability in dialysis frequency, with 98.7% of participants attending three times per week. The lack of significant predictors for the physical and social domains suggests that clinical dialysis parameters alone are insufficient to explain the complex variations in patients’ lived experiences.

The clinical HD profile of this cohort also warrants comparison with other studies. The high burden of comorbidities in our sample is consistent with reports from regional and international HD populations, in which hypertension, diabetes, and cardiovascular disease frequently coexist and contribute to lower QOL and greater symptom burden (Ajeebi et al., 2020; Ishtawi et al., 2023; Al Salmi et al., 2021). Likewise, the finding that nearly all participants underwent HD three times weekly reflects the standard maintenance-HD schedule described in Saudi and neighboring settings, which may explain why weekly frequency showed statistical significance but limited practical interpretability in this sample because there was almost no variability in exposure (Al Wakeel et al., 2012; Samoudi et al., 2021). More than half of our participants had been receiving HD for 1–6 years, suggesting an established treatment experience; however, similar to previous studies, dialysis duration alone did not appear sufficient to explain broader differences in QOL when compared with psychosocial and socioeconomic influences (Kefale et al., 2019; Alshehri et al., 2023). Finally, the very high proportion of participants who did not know their CKD stage is clinically important. Although direct comparison is difficult because many HD studies classify patients simply as having end-stage kidney disease rather than asking for self-reported stage, this finding may indicate limited disease-specific knowledge and highlights the need for better patient education within dialysis care (Gebrie et al., 2023; Upadhyay et al., 2014).

### Limitations

Several limitations should be considered. The study employed a cross-sectional design with convenience sampling at a single center, limiting causal inference and generalizability. Pain was measured using a self-report scale during the hemodialysis session, which may not capture the fluctuating nature of chronic pain between sessions. Additionally, we lacked detailed data on specific pain sources, analgesic medication use, vascular access type, and dialysis adequacy. The assistance provided by research assistants for questionnaire completion introduces a possible response bias. Finally, a very large proportion of participants (86%) were unaware of their specific CKD stage, limiting the utility of disease severity in our analysis.

### Implications for nursing practice

The findings highlight critical opportunities for nurses to improve the QOL of adult HD patients. In clinical practice, nurses should prioritize comprehensive, culturally sensitive pain management strategies to mitigate the negative impact of pain on physical and psychological well-being. Furthermore, the high rate of patients unaware of their CKD stage underscores a pressing need for improved health literacy and self-management education. Nurses should embed age-appropriate psychosocial support and work to connect low-income patients with available socioeconomic resources to holistically address QOL disparities.

## Conclusions

Pain is a prevalent and significant correlate of reduced quality of life among adult hemodialysis patients in Saudi Arabia. While clinical factors play a minor role, socioeconomic status and pain severity are robust predictors of patient well-being, highlighting the need for holistic interventions that integrate culturally sensitive pain management and socioeconomic support.

## Supplemental Information

10.7717/peerj.21892/supp-1 Supplemental Information 1 Descriptive statistics for the WHOQOL-BREF questionnaire.

10.7717/peerj.21892/supp-2 Supplemental Information 2 Raw dataset with codebook.

10.7717/peerj.21892/supp-3 Supplemental Information 3 STROBE checklist.

## Additional Information and Declarations

### Data Availability

The following information was supplied regarding data availability:

The raw measurements are available in the Supplemental Files.
